# Comparing the clinical efficacy of three surgical methods for cesarean scar pregnancy

**DOI:** 10.1186/s12905-023-02415-y

**Published:** 2023-05-17

**Authors:** Shaoying Zeng, Yang Wang, Ping Ye, Ling Xu, WenLing Han, Feng Li, Chen Tang, Jieli Zhou, Xiaoying Xie

**Affiliations:** 1 Department of Obstetrics and Gynecology, The First Affiliated Hospital of Jinan University, 510630 Guangzhou, Guangdong China; 2 Department of Obstetrics and Gynecology, The First Affiliated Hospital of Gannan Medical University, 34100 Ganzhou, Jiangxi China; 3 Department of Obstetrics and Gynecology, The First Affiliated Hospital of Gannan Medical University, No.128 Jin Ling Road, Ganzhou, Jiangxi, 34100 China

**Keywords:** Cesarean section scar pregnancy, Hysteroscopy, Ultrasonic monitoring, Uterine curettage

## Abstract

**Background:**

We aimed to compare the clinical efficacy of three surgical methods in the treatment of various types of cesarean scar pregnancy (CSP).

**Methods:**

Herein, 314 cases of CSP were treated in the department of Obstetrics and Gynecology of the First Affiliated Hospital of Gannan Medical University between June 2017 and June 2020. The patients were divided into three groups based on the treatment received: group A (n = 146; curettage by pituitrin combined with ultrasonic monitoring and hysteroscopy-guided surgery), group B [n = 90; curettage after methotrexate (MTX) injection into the local gestational sac], and group C (n = 78; laparoscopic, transvaginal, and transabdominal cesarean scar resection). These groups were divided into three subgroups (type I, type II, and type III) according to the CSP type of the patients.

**Results:**

The intraoperative blood loss, length of hospital stay, hospitalization cost, menstrual recovery time, and serum β-HCG normalization time were lower in groups A than in groups B or C with type I, II and III CSP (P < 0.05). Operative efficiency and Successful second pregnancy rate were higher in groups A than in groups B or C with type I and II CSP (P < 0.05). But in type III CSP, the complications were more serious in group A than group C.

**Conclusions:**

Curettage by pituitrin combined with ultrasonic monitoring and hysteroscopy-guided surgery is an effective and relatively safe treatment for patients with type I and II CSP. Laparoscopic surgery is more suitable for type III CSP.

**Supplementary Information:**

The online version contains supplementary material available at 10.1186/s12905-023-02415-y.

## Introduction

Cesarean scar pregnancy (CSP) is a condition wherein the embryo gets implanted into the myometrial defect at the site of the scar left by the previous cesarean delivery; these pregnancy tissues need to be removed early in the pregnancy (gestational age < 12 weeks) and pose the risk of potential iatrogenic dangerous consequence if the intervention is delayed. The exact incidence of CSP in China is not clear. According to foreign literature, the incidence of CSP has been reported as approximately 1:2000 of all pregnancies [1, 2]. In recent years, the detection rates of CSP have improved with advancements in and wide application of vaginal ultrasound, magnetic resonance imaging, and other medical diagnostic technologies; these techniques have allowed clinicians to consider CSP in their differential diagnoses for several cases [3]. Patients with CSP may have no symptoms or only a little vaginal bleeding, with no obvious specific manifestations in the early stages of pregnancy. According to reports, at least 13.6% of patients with CSP in early pregnancy are misdiagnosed [4]. Therefore, women with a history of cesarean section should be examined by vaginal color ultrasound for an early diagnosis to determine the position of the embryo implant and identify whether it is growing inside the myometrium or on the fibrous tissue of the previous of the cesarean scar.

In 2016, to aid better diagnosis and treatment of CSP, the Family Planning Group, the Chinese Medical Society of Obstetrics and Gynecology Expert Consensus on Diagnosis and Treatment of Cesarean Section Scar Pregnancy [5], which has been previously described in detail [6], CSP was classified into three types (Supplementary Table 1 ) based on ultrasound findings of gestational sac implantation into the myometrial defect along the anterior wall caused by a prior cesarean delivery, the direction of growth of the pregnancy sac, blood flow features, and the thickness of the myometrium between the implanted pregnancy sac and the bladder wall (myometrial thickness). The use of different classification methods can affect the choice of clinical treatment and the treatment effect. In this study, we compare the clinical efficacy of three different treatment methods for CSP and explore the safety and efficacy of the use of curettage by pituitrin combined with ultrasonic monitoring and hysteroscopy-guided surgery for CSP treatment.

## Materials and methods

### General data

314 patients who visited the Department of Obstetrics and Gynecology of the First Affiliated Hospital of Gannan Medical University from June 2017 to June 2020 and met the inclusion criteria. The age, gravidity, parity, number of previous CS, gestational age, time of interval between the last pregnancy and current CSP, number of abortions, thickness of the cesarean incision scar, and serum β-HCG levels were analyzed by reviewing the medical records. The differences in the general situation were not statistically significant among the groups A, B, and C (*P* > 0.05). (Supplementary Table 2 )

The patients were divided into three groups based on the treatment method used: group A (curettage by pituitrin combined with ultrasonic monitoring and hysteroscopy-guided surgery), group B (curettage after methotrexate (MTX) injection into the local gestational sac), and group C (laparoscopic, transvaginal, and transabdominal cesarean scar resection). Each of these groups was divided into three subgroups based on the type of CSP identified by ultrasonography. In group A (n = 146), 64, 77, 5 cases of type I, II, and III CSP, respectively. In group B (n = 90), there were 42 cases of type I CSP, 48 cases of type II CSP, and no type III CSP cases. In group C (n = 78), 26, 37, and 15 cases of type I, II, and III CSP, respectively.

## Diagnosis

The diagnosis of CSP mainly depends on transvaginal sonography (TVS) with a positive pregnancy test. The diagnostic criteria were as follows: (1) No pregnancy tissue was found in the uterine cavity or the cervical canal; (2) Gestational sac was visible at the site of the scar from previous cesarean incision; (3) The muscular layer of the anterior uterine wall lacked continuity, and the muscular layer of the uterus between the pregnancy sac and the bladder appeared evidently thin, elongated, or absent; and (4) Color Doppler ultrasonography (CDFI) revealed high-speed and low-resistance blood flow signals around the pregnancy sac or mass. (5) Patients with a gestational period of < 12 weeks and stable vital signs.

## Methods

### Group A: Curettage by pituitrin combined with ultrasonic monitoring and hysteroscopy-guided surgery

The venous passage was established, and the other rescue equipment was prepared. Patients were administered a cervical injection of pituitrin 6 U and underwent curettage with ultrasonic monitoring performed by experienced gynecologists and sonographers. Pregnancy tissues were then sent for pathological examination. Hysteroscopy (Bettocchi office Hysteroscope “size 4.3” Karl Storz, Germany) was performed again to observe whether the gestational sac removal was clean and whether the surgical wound was bleeding. If residual pregnancy tissue or bleeding was identified, hysteroscopic removal could be performed. More bleeding can be stopped by pressing a three-chamber catheter.

### Group B: Curettage after methotrexate (MTX) injection into the local gestational sac under ultrasound guidance

This involved ultrasound-guided injection of 50 mg/m^2^ MTX into the gestational sac through the cervix using a puncture needle. When serum β-HCG levels were down to normal levels or low levels and ultrasound showed no obvious blood flow, uterine curettage was performed under ultrasound guidance. If obvious active bleeding was noted, further bleeding was stopped by pressing a three-chamber catheter. The tissue was sent for pathological examination.

### Group C: laparoscopic, transvaginal or transabdominal cesarean scar resection

Removal of pregnancy tissues from the uterine scar was performed by transvaginal or transabdominal resection or by laparoscopic surgery, removing the pregnancy tissue, trimming the old tissue, and suturing the uterine incision. The tissue was sent for routine pathological examination.

The information on following aspects was collected and summarized: intraoperative bleeding, operative time, operative efficiency, length of hospital stay, hospitalization cost, serum β-HCG normalization time, menstrual recovery time, pregnancy again and successful second pregnancy n (%). The evaluation criteria of total operative efficiency are shown in Supplementary Table 3 .

### Statistical analysis

All data were statistically analyzed by SPSS 24.0 software. As the measurement data were not normally distributed, the median (P25, P75) was used to represent the measurement data. A non-parametric test was used for between-group comparisons, and if multiple group comparisons were statistically significant, multiple comparisons were performed. Enumeration data were expressed as frequency (%), and chi-square test was used for differences between groups. *P* < 0.05 was considered statistically significant.

## Results

### Comparison of indices of groups A1, B1, and C1

The intraoperative blood loss was significantly lower in group A1 than in group B1 (*P* < 0.05); The operative time was significantly shorter in groups A1 and B1 than in group C1 (*P* < 0.05). The operative efficiency was significantly higher in groups A1 and C1 than in group B1, with statistically significant difference (*P* < 0.05; effective rates are shown in Table 1). The length of hospitalization was significantly shorter in group A1 than group C1 and shorter than group B2; Hospitalization cost was significantly lower in groups A1 and B1 than in group C1 (*P* < 0.05). Recovery times were significantly shorter in groups A1 and C1 than in group B1 (*P* < 0.05). Specific results are shown in Table 1.

**Table 1:** Comparison among groups A1, B1, and C1

Observational index	A1 (n = 64)	B1 (n = 42)	C1 (n = 26)	H	P
Intraoperative blood loss (mL)	20 (10, 50)	50 (30, 100)	25 (20, 52.5)	25.283	< 0.001
Operative time (min)	25 (18.5, 39)	32.5(30, 40)	84 (60, 108.5)	62.530	< 0.001
Operative efficiency n (%)	64 (100)	36 (86)	25 (96)	9.558	0.003#
Length of hospitalization (days)	1 (1, 2)	9.5(5, 15.25)	3 (1.75, 3)	85.515	< 0.001
Hospitalization cost ($)	824(735, 903)	832(638, 1112)	1670(1335, 2035)	55.600	< 0.001
Menstrual recovery time (days)	32.5 (29, 37)	49.5(43.5, 60)	34 (30.75, 38)	51.824	< 0.001
β-hCG recovery time (days)	30 (16.25, 31)	39(31.5, 44)	30(27.5, 32.25)	34.412	< 0.001
Pregnancy again n (%)	16 (25)	7 (16.67)	6 (23.07)	1.050	0.591
Successful second pregnancy n (%)	16 (100)	3 (42.86)	5 (83.33)	5.460	0.065

#: Fisher’s exact test

### Comparison of indexes of each type in groups A2, B2, and C2

The intraoperative blood loss was significantly greater in groups B2 and C2 than in group A2 (*P* < 0.05). Pairwise comparisons showed that the operative time was significantly shorter in groups A2 and B2 than in group C2 (*P* < 0.05). The operative efficiency was significantly higher in groups A2 and C2 than in group B2 (*P* < 0.05; effective rates are shown in Table 2). The length of hospitalization was significantly shorter in groups A2 and C2 than in group B2. Hospitalization cost in group A2 was lower than group B2 and lower than group C2, and the differences were statistically significant (*P* < 0.05). These recovery times were significantly shorter in group A2 than group C2 and shorter than group B2 (*P* < 0.05). However, the outcomes of the second pregnancy significantly differed among the three groups, with group A2 having the best outcome, group C2 having intermediate outcomes, and group B2 having the worst outcome, and the differences were statistically significant (*P* < 0.05). Specific results are shown in Table 3.

**Table 2:** Comparison between groups A3 and C3

Observational index	A3 (n = 5)	C3 (n = 15)	Z	P
Intraoperative blood loss (mL)	400 (325, 1000)	300 (200, 600)	-1.321	0.187
Time of operation (min)	164 (62.5, 233.5)	164 (120 ,200)	-0.349	0.727
Surgical treatment n (%)	3 (60)	12 (80)		0.560#
Length of hospitalization (days)	5 (3.5, 7.5)	9 (6, 12)	-2.233	0.026
Hospitalization cost ($)	799 (541, 1904)	2493 (1666, 3167)	-2. 488	0.013
Menstrual recovery time(days)	30 (24, 39.5)	33 (30, 40)	-0.789	0.430
β-HCG recovery time (days)	39 (10.5, 41)	33 (29, 37)	-0.131	0.896

#: Fisher’s exact test

**Table 3:** Comparison among groups A2, B2, and C2

Observational index	A2(n = 77)	B2 (n = 48)	C2 (n = 37)	H	P
Intraoperative blood loss (mL)	30 (20, 50)	150(100, 197.5)	120 (50, 200)	68.579	< 0.001
Operative time (min)	32 (25.5, 40)	39 (30, 43.75)	130(69, 159.5)	82.956	< 0.001
Surgical treatment n (%)	73 (94.80)	31 (64.58)	36 (97.30)	27.846	< 0.001
Length of hospitalization (days)	5 (4, 8.5)	20.5 (15, 26)	6 (5, 9)	66.435	< 0.001
Hospitalization cost ($)	865(775, 1022)	1132(813, 1358)	2180(1803, 2663)	84.770	< 0.001
Menstrual recovery time	31 (29, 34)	47.5 (39, 60)	35 (32, 38)	59.045	< 0.001
(days)					
β-hCG recovery time (days)	29 (27, 31)	39 (33.25, 44)	30 (28, 33.5)	44.144	< 0.001
Pregnancy again n (%)	12 (15.58)	3 (6.25)	4 (10.81)	2.527	0.283

#: Fisher’s exact test

### Comparison of various types of indexes in groups A3 and C3

The two groups did not differ in terms of intraoperative blood loss and operative time (*P* < 0.05). Specific results are shown in Table 2. But in type III CSP, the complications were more serious in group A than in group C, and 3 of the 5 patients with type III had intraoperative hemorrhage and were transferred to laparotomy or laparoscopic surgery.

No statistically significant difference was identified in operative efficiency between the two groups (*P* > 0.05; the criteria of operative efficiency are shown in Supplementary Table 3 ). The length of hospitalization and hospitalization cost were significantly lower in group A3 than in group C3 (*P* < 0.05). Specific results are shown in Table 2.

No significant between-group differences were identified in terms of menstrual recovery time and postoperative serum β-HCG recovery time (*P* > 0.05). Specific results are shown in Table 2.

## Discussion

CSP is a special type of ectopic pregnancy wherein the gestational sac gets implanted in and grows at the myometrial defect or the scar left behind in the uterine cavity wall after the last cesarean section; however, CSP differs from ectopic pregnancy in that the embryo implants and grows inside the myometrium and the fibrous tissue of the previous cesarean scar. This situation greatly increases the risk of critical maternal complications, such as uterine perforation and massive bleeding. The condition is also complicated by the difficulties associated with establishing a timely diagnosis and determining the best treatment.

Clinical manifestations of CSP are often ranging from no symptoms to uterine rupture and massive bleeding. According to the statistics of the Society for Maternal-Fetal Medicine (SMFM) guidelines in 2020 [7], no symptoms and painless vaginal bleeding each accounted for about 1/3 of the clinical manifestations of CSP, and pain with or without bleeding was reported in ~ 25% of the cases. CSP in patients is generally detected during their first trimester visit, and most CSP patients are reportedly diagnosed at 5–9 weeks [8]. If CSP is misdiagnosed or missed and continues to develop into the second and third trimesters, serious complications can occur, and the patients are at high risk of perforation and massive bleeding, which can severely threaten their reproductive function and life. Therefore, for pregnant women having undergone a cesarean section during their previous pregnancy, specific attention to early ultrasound findings is needed to ensure that CSP is avoided. Ultrasound can effectively diagnose CSP early in the pregnancy. Ultrasound examination can determine the status of pregnancy tissues in patients and is instrumental in assisting CSP typing [9].

Since the first international report on CSP by Larsen et al. [10] in 1978, more than 30 clinical treatment methods for CSP have been established, and these methods can be broadly divided into medical (drugs) and surgical intervention. Due to the long treatment cycle, poor efficacy and safety, and numerous adverse reactions, drugs have not been routinely recommended [11]. Surgery is the preferred treatment for CSP. Clinical surgical methods mainly include uterine curettage, hysteroscopic surgery, laparotomy, laparoscopic surgery, vaginal surgery, uterine artery embolization, high-intensity focused ultrasound, robotic surgery, and a combined UAE–hysteroscopic laser surgery [12, 13].

The most appropriate treatment for CSP should be determined as per the specific situation of the patient. Due to the complexity of and risks associated with CSP, according to SMFM [7], expectant management for CSP is not recommended. Expectant management of a cesarean scar pregnancy is associated with a high risk of hysterectomy due to a morbidly adherent placenta [14]. Pregnancy should be terminated early, and the pregnancy tissues should be surgically removed to avoid life-threatening serious complications and preserve the reproductive function of the mother. Medical intervention to terminate CSP involves systemic drug therapy, local drug therapy, and combination drug therapy In several studies [15–17], 25% of patients receiving MTX still require additional treatment, and ~ 13% of patients receiving MTX developed serious complications. A study of 101 individuals with cesarean scar pregnancy treated with an ultrasound-guided methotrexate injection reported a mean β-HCG normalization time of 40 ± 14 days (range: 21–140 days) [18]. Our study of cases with type I and type II CSP treated with an ultrasound-guided curettage after methotrexate (MTX) injection showed that the median time to β-HCG normalization was 39 (range: 31.5–44) and 39 (range: 33.25–44) days, respectively, which was consistent with the report [18].

At present, there are many clinical treatment methods for CSP; however, there is no unified treatment standard. A literature review [19] reported that the different therapeutic effects of different CSP treatments could be associated with different types of CSP. Therefore, CSP classification should be determined before surgery, and treatment should be selected according to the type of CSP.

Our study comprised 132 cases of type I, 162 cases of type II, and 20 cases of type III CSPs. Three methods (curettage by pituitrin combined with ultrasonic monitoring and hysteroscopy-guided surgery, curettage after methotrexate (MTX) injection into the local gestational sac, laparoscopic, vaginal, or transabdominal surgery for cesarean scar resection) were used in the CSP patients.

For type I and II CSP, curettage by pituitrin combined with ultrasonic monitoring and hysteroscopy-guided surgery was more suitable. Pituitrincan rapidly and effectively shrink small arteries, capillaries, and even uterine smooth muscles. Rational and effective application of pituitrin can significantly reduce intraoperative bleeding, relieve patients’ surgical trauma, and reduce and prevent intraoperative complications. Curettage under ultrasonography, the tissues could be visually identified. This can avoid extensive damage to the endometrium caused by curettage [20], to protect the fertility. Hysteroscopic removal of CSP has been reported to be safe and effective as an alternative minimally invasive surgery [19]. Hysteroscopy provides clear visual access to the scar, making it easy to identify the range of affected tissues and resect the pregnancy tissue [12]. Our data showed that intraoperative bleeding, recovery time, postoperative hospital stay, the operative time and hospitalization cost were shorter and menstrual, β-HCG normalized faster in group A than in group B or C. Furthermore, operative efficiency was higher in group A than in group B or C. Group B had inferior outcomes in terms of intraoperative blood loss, length of hospital stay, menstrual recovery, and β-HCG normalization time. The operative processes of the three groups were successful, and no postoperative tissue residue or serious complications caused by intrauterine adhesions and other complications were noted.

For type III CSP, cesarean scar resection is safer. Zeller et al. [21] recommended scar tissue removal as the first choice for patients with high-risk factors. Our data showed that group A and C did not differ in the amount of intraoperative blood loss, operative time, operative efficiency, length of hospitalization, and hospitalization cost, but the complications were more serious in group A than in group C, and 3 of the 5 patients with type III CSP had intraoperative hemorrhage and were transferred to laparotomy or laparoscopic surgery. The result was in accordance with the studies [3], which found that laparoscopic cesarean scar resection (LCSR) were associated with a high success rate (95.5–97.1%) and no major complications for all types of CSP. However, the type I and II patients choose laparoscopic, transvaginal, or transabdominal cesarean scar resection, which increased hospitalization costs. So, we propose that laparoscopic, transvaginal, and transabdominal cesarean scar resection approaches are more suitable for type III CSP as they can reduce the operation risk, such as reducing massive bleeding during operation, and prevent uterine perforation. However, it costed much more than curettage by pituitrin combined with ultrasonic monitoring and hysteroscopy-guided surgery and needed longer hospitalization.

## Conclusions

Curettage by pituitrin combined with ultrasonic monitoring and hysteroscopy-guided surgery is an effective and relatively safe treatment option for patients with type I and II CSP. But laparoscopic, transvaginal, transabdominal cesarean scar resection is more suitable for type III CSP.

## Electronic supplementary material

Below is the link to the electronic supplementary material.

Additional File 1:

## Data Availability

All data generated or analyzed during this study are included in this article. Further enquiries can be directed to the corresponding author.

## Abbreviations

CSP: cesarean scar pregnancy
MTX: methotrexate
CDFI: Color Doppler ultrasonography
LCSR: laparoscopic cesarean scar resection
