# Supplementary material for: Comparing the clinical efficacy of three surgical methods for cesarean scar pregnancy
Source: BMC Womens Health. 2023 May 17;23:271. doi: 10.1186/s12905-023-02415-y (PMC10193701; doi:10.1186/s12905-023-02415-y)
Supplement: Supplementary file 1 — Additional File 1: [file 12905_2023_2415_MOESM1_ESM.docx]

**Supplementary materials**

**Introduction**

In 2016, to aid better diagnosis and treatment of CSP, the Family Planning Group, the Chinese Medical Society of Obstetrics and Gynecology Expert Consensus on Diagnosis and Treatment of Cesarean Section Scar Pregnancy [5], which has been previously described in detail [6], CSP was classified into three types (Supplementary Table 1) based on ultrasound findings of gestational sac implantation into the myometrial defect along the anterior wall caused by a prior cesarean delivery, the direction of growth of the pregnancy sac, blood flow features, and the thickness of the myometrium between the implanted pregnancy sac and the bladder wall (myometrial thickness). The use of different classification methods can affect the choice of clinical treatment and the treatment effect. In this study, we compare the clinical efficacy of three different treatment methods for CSP and explore the safety and efficacy of the use of curettage by pituitrin combined with ultrasonic monitoring and hysteroscopy-guided surgery for CSP treatment.

Supplementary Table 1. CSP classification according to the Family Planning Group, the Chinese Medical Society of Obstetrics, and Gynecology Expert Consensus on Diagnosis and Treatment of Cesarean Section Scar Pregnancy

| Type | Diagnostic standards |
| --- | --- |
| I | (1) Gestational tissue was partially implanted in the uterine scar, partially or mostly located in the uterine cavity, and some even reached the bottom of the uterine cavity;  (2) The gestational sac was obviously deformed, elongated, and the lower end was acute;  (3) The myometrium between the gestational sac and the bladder was >3 mm;  (4) CDFI: trophoblast blood flow signal (low resistance blood flow) was seen in the scar. |
| II | (1) Gestational tissue was partially implanted in the uterine scar, partially or mostly located in the uterine cavity, and some even reached the bottom of the uterine cavity;  (2) The gestational sac was obviously deformed, elongated, and the lower end was acute;  (3) The myometrium between the gestational sac and the bladder was≤3 mm;  (4) CDFI: trophoblast blood flow signal (low resistance blood flow) was seen in the scar. |
| III | (1) Gestational tissues were completely implanted in the muscular layer of the uterine scar and protruded outward to the bladder;  (2) Emptiness of the uterine cavity and cervical canal;  (3) The myometrium between the gestational sac and the bladder was significantly thinner or even missing, the thickness was ≤3 mm;  (4) CDFI: trophoblast blood flow signal (low resistance blood flow) was seen in the scar. |

**1. Data and methods**

**1.1** **General data**

314 patients who visited the Department of Obstetrics and Gynecology of the First Affiliated Hospital of Gannan Medical University from June 2017 to June 2020 and met the inclusion criteria. The age, gravidity, parity, number of previous CS, gestational age, time of interval between the last pregnancy and current CSP, number of abortions, thickness of the cesarean incision scar, and serum β-HCG levels were analyzed by reviewing the medical records (Supplementary Table 2). The differences in the general situation were not statistically significant among the groups A, B, and C (*P* > 0.05).

Supplementary Table 2. Comparison of the general situation of three groups A, B and C

| Characteristics | A (n = 146) | B (n = 78) | C (n = 90) | *F* | *P* |
| --- | --- | --- | --- | --- | --- |
| Age (years) | 33 ± 5.103 | 33.02 ± 4.839 | 32.41 ± 4.911 | 0.423 | 0.655 |
| Gravidity | 3.95 ± 1.433 | 3.89 ± 1.487 | 3.65 ± 1.337 | 1.094 | 0.336 |
| Gestational age (days) | 48.51 ± 10.67 | 50.17 ± 10.174 | 48.04 ± 10.884 | 1.002 | 0.368 |
| Parity | 1.78 ± 0.719 | 1.78 ± 0.595 | 1.96 ± 0.692 | 2.103 | 0.124 |
| Number of abortions | 1.97 ± 1.315 | 1.81 ± 1.373 | 1.67 ± 1.101 | 1.431 | 0.241 |
| Number of previous cesareans | 1.50 ± 0.590 | 1.62 ± 0.610 | 1.54 ± 0.596 | 1.170 | 0.312 |
| Time interval between last pregnancy and CSP (years) | 4.990 ± 1.669 | 5.237 ± 1.624 | 4.80 ± 2.26 | 1.229 | 0.294 |
| Thickness of cesarean incision scar (mm) | 3.299 ± 2.332 | 3.317 ± 2.123 | 3.264 ± 2.183 | 0.012 | 0.988 |
| Blood β-hCG (mlU/mL) | 36558.18 ± 39371 | 36464.39 ± 38944 | 37953.94 ± 40980 | 0.038 | 0.962 |

**2. Methods**

The information on following aspects was collected and summarized: intraoperative bleeding, operative time, operative efficiency, length of hospital stay, hospitalization cost, serum β-HCG normalization time, menstrual recovery time, pregnancy again and successful second pregnancy n (%). The evaluation criteria of total operative efficiency are shown in Supplementary Table 3.

Supplementary Table 3. Evaluation criteria of surgical efficacy

| Excellent | The pregnancy tissue was completely cleared and the serum β-HCG level normalized. |
| --- | --- |
| [Effective](javascript:;) | The pregnancy tissue was not completely cleared, but the serum β-HCG level normalized, albeit slowly. |
| Ineffective | The removal of the pregnancy sac failed, and the serum β-HCG level remained high, initially declined and then increased, or never normalized. The patient was transferred or re-operated due to uterine perforation and massive bleeding. |
| Total efficacy rate (%) | (Excellent + [Effective](javascript:;))/total number of cases in this group × 100% |
